# Contrariety in Surgical Practice

**Published:** 1817-03

**Authors:** 


*For the London Medical and Physical Journal.*

*Contrariety in Surgical Practice;* by A VALETUDINARIAN.

THERE is scarcely a profession wherein there exists a greater difference of opinion, so far as regards the practical part of it, than in that of surgery, especially as to the treatment of what is termed Cancer. It is a complaint which, by most surgeons, has been deemed incurable, except by *extirpation* by the knife; and then the disease has been frequently known to recur. Two gentlemen have obtained considerable notoriety by their modes of treating this disease. The method of the one, however, is diametrically opposite to that of the other; but, which of the two is to be preferred, I must leave to the consideration of their brethren and the public, or rather to their patients. While the one removes the diseased part by *extraction*, by an application of his own,—the other, equally singular, gets rid of the cancer by means of *pressure* made on the part affected; or, in other words, the one draws *out* the tumour or ulcer, when the other presses it *inward*. Both these gentlemen stand high in the profession, and have followed their several modes for some years—the first about ten, and the latter about three, years. The one supposes, that, by pressing the part till the whole becomes absorbed, the person is thereby cured;—while the other carefully avoids all pressure, making his application to draw out the diseased substance only, and permits the cavity to heal to its natural level—his patient likewise recovers.

It is not my intention, Mr. Editor, at present, to offer any remarks on either of these gentlemen's practice, further than to repeat that they are, unquestionably, very opposite to each other.

*London; Feb.* 2, 1817.

---

**We are much obliged to this gentleman for his remarks; and, if he will pursue them systematically, we hope it may lead to a more accurate discrimination of the diseases so generally confounded under the term Cancer.—EDIT.**

